# Supplementary material for: Causal inference on neuroimaging data with Mendelian randomisation
Source: Neuroimage. 2022 Sep;258:119385. doi: 10.1016/j.neuroimage.2022.119385 (PMC10933777; doi:10.1016/j.neuroimage.2022.119385)
Supplement: Supplementary Data S2 — Supplementary Raw Research Data. This is open data under the CC BY license http://creativecommons.org/licenses/by/4.0/ [file mmc2.pdf]

## Supplementary Material

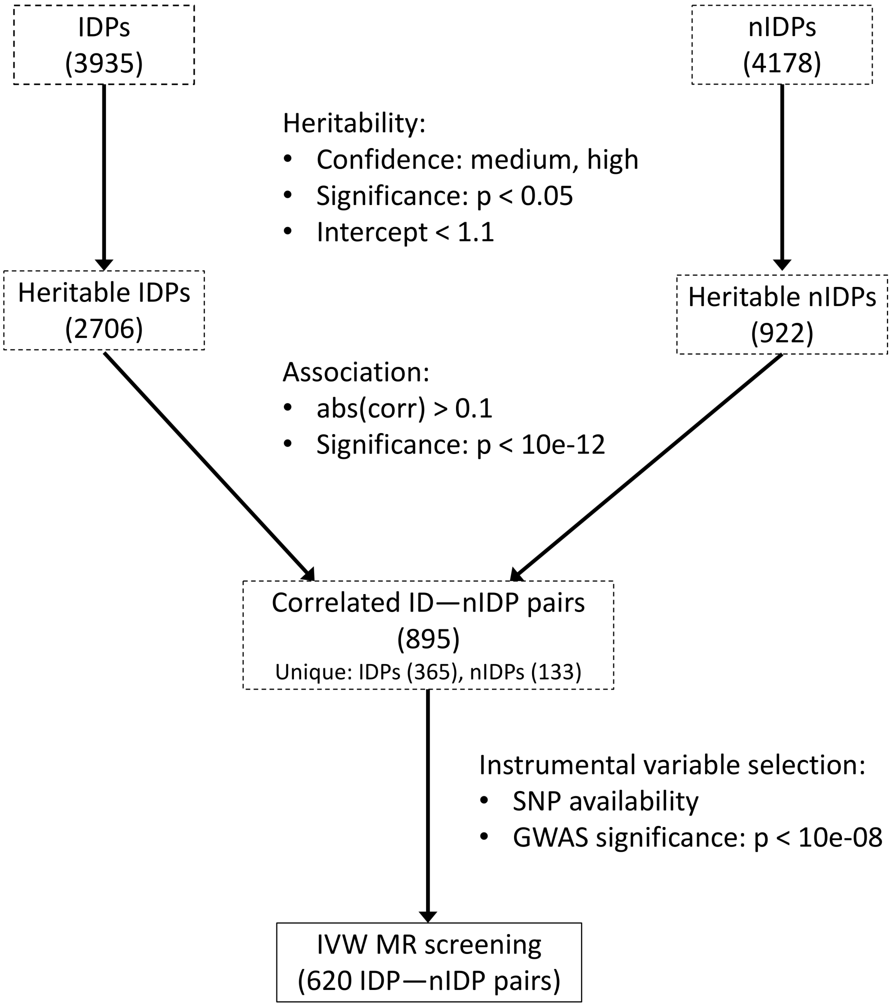


Figure 12: Flowchart showing the pre-selection and screening procedures of potential causal variable pairs as described in Subsections 3.1.1 and 3.1.2.

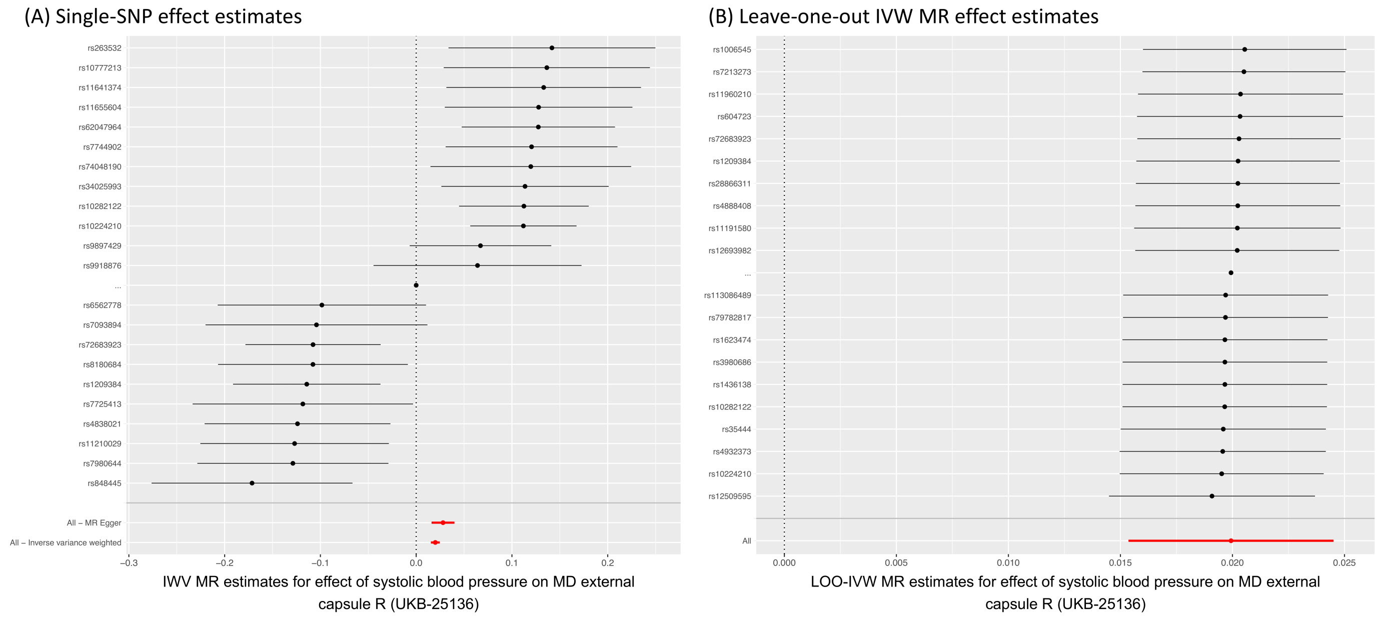


Figure 13: MR sensitivity analysis of the causal effect of systolic blood pressure on mean diffusivity of the external capsule WM tract R (UKB-ID 25316). (A) Single-SNP ratio estimates. (B) Leave-one-out MR results. Only results for 20 SNPs with the most extreme effect estimates are shown. Errorbars indicate 95% confidence intervals.

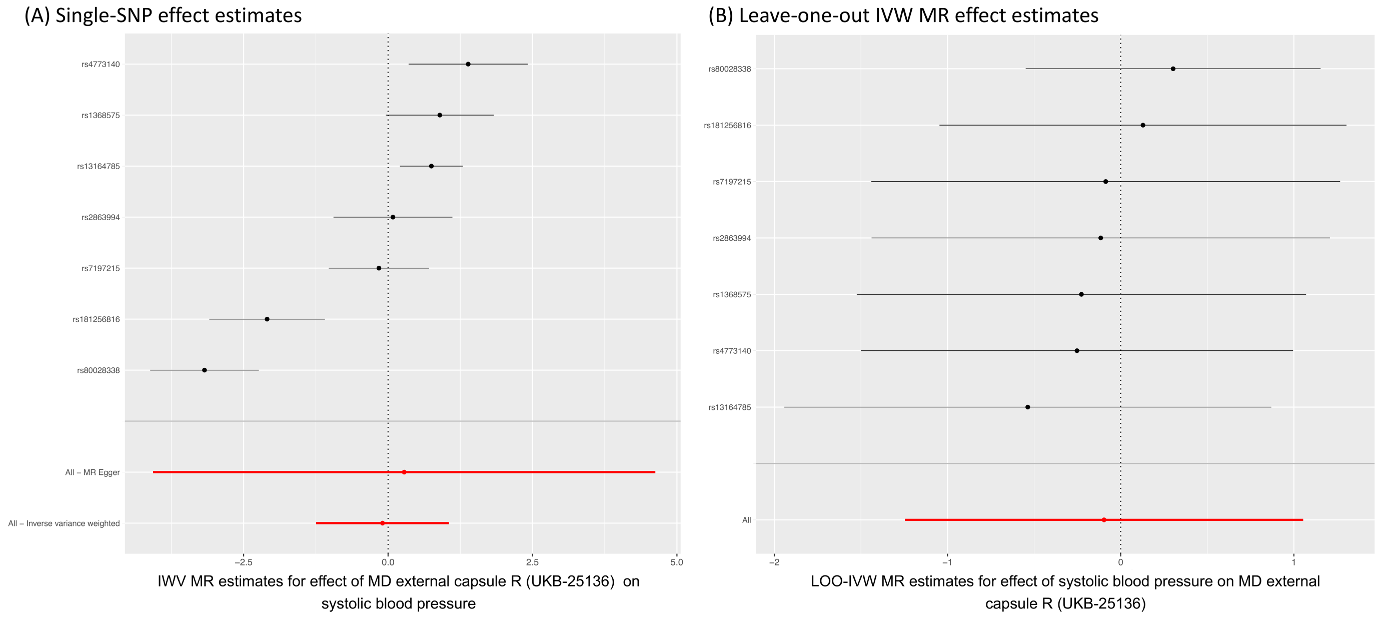


Figure 14: MR sensitivity analysis of the causal effect of mean diffusivity of the external capsule WM tract R (UKB-ID 25316) on systolic blood pressure. (A) Single-SNP ratio estimates. (B) Leave-one-out MR results. Errorbars indicate 95% confidence intervals.

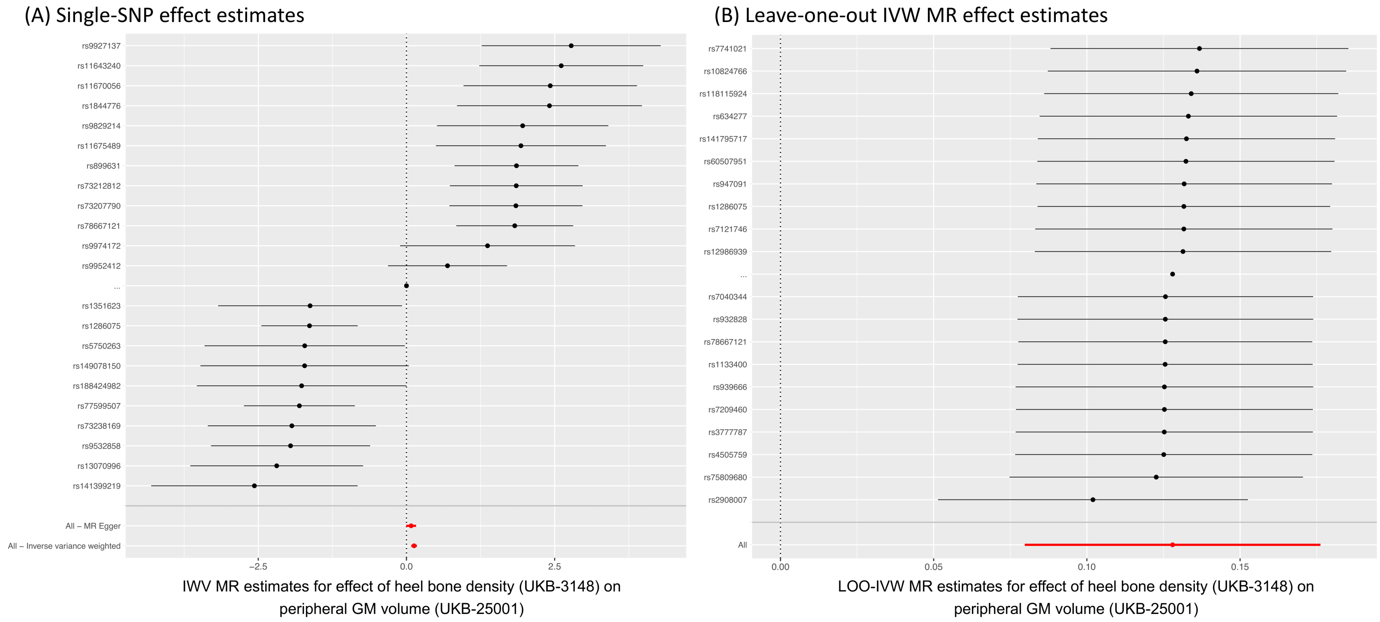


Figure 15: MR sensitivity analysis of the causal effect of heel bone density (UKB-ID 3148) on peripheral grey matter volume (UKB-ID 25001). (A) Single-SNP ratio estimates. (B) Leave-one-out MR results. Only results for 20 SNPs with the most extreme effect estimates are shown. Errorbars indicate 95% confidence intervals.

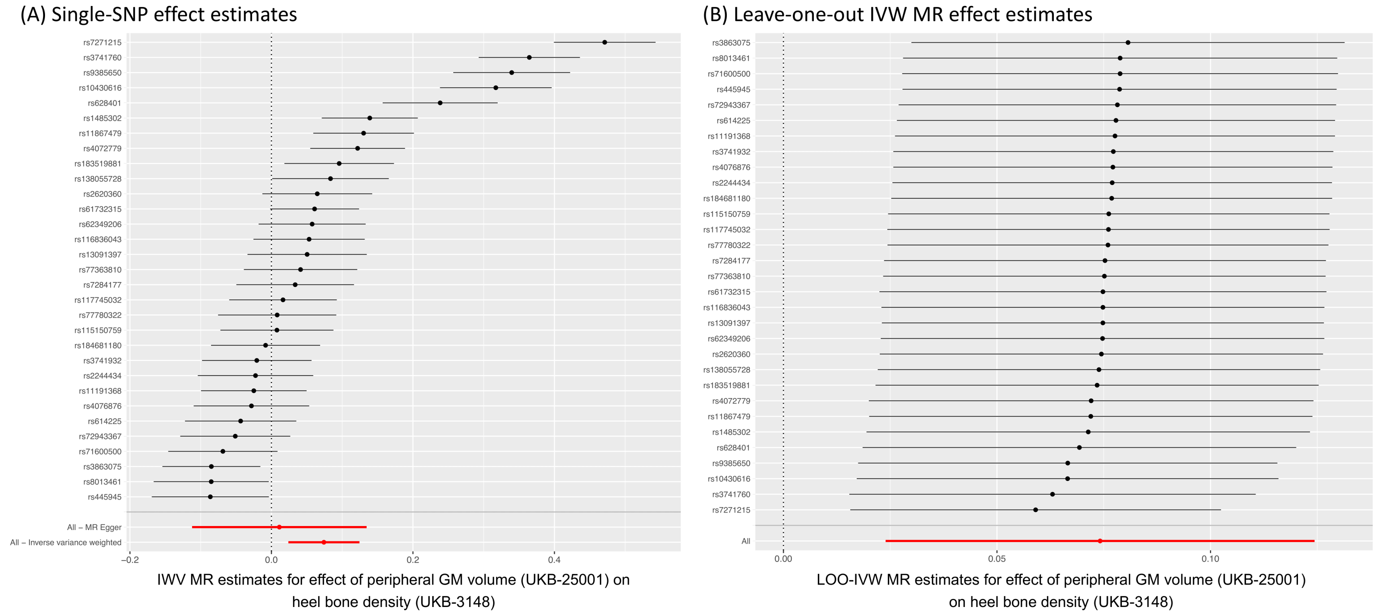


Figure 16: MR sensitivity analysis of the causal effect of mean diffusivity of peripheral grey matter volume (UKB-ID 25001) on heel bone density (UKB-ID 3148). (A) Single-SNP ratio estimates. (B) Leave-one-out MR results. Errorbars indicate 95% confidence intervals.

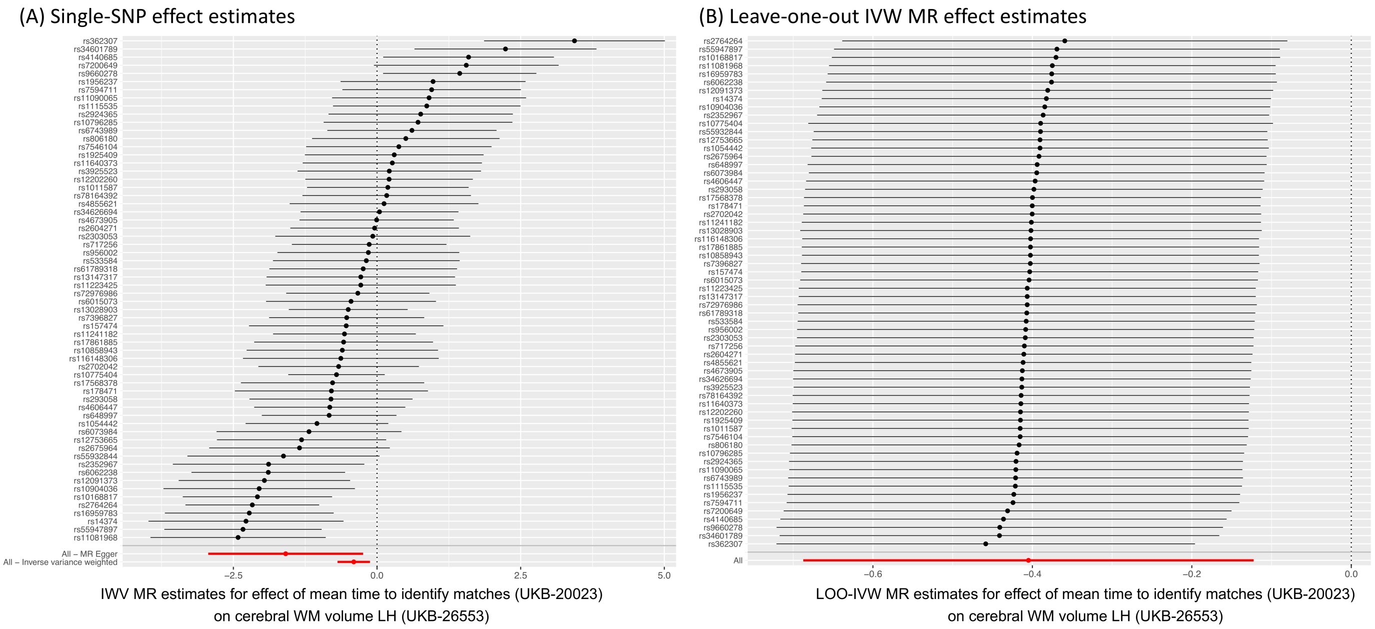


Figure 17: MR sensitivity analysis of the potentially causal effect of correctly identifying matches (UKB-ID 20023) on cerebral white matter volume (UKB-ID 26553). (A) Single-SNP ratio estimates. (B) Leave-one-out MR results. Errorbars indicate 95% confidence intervals.

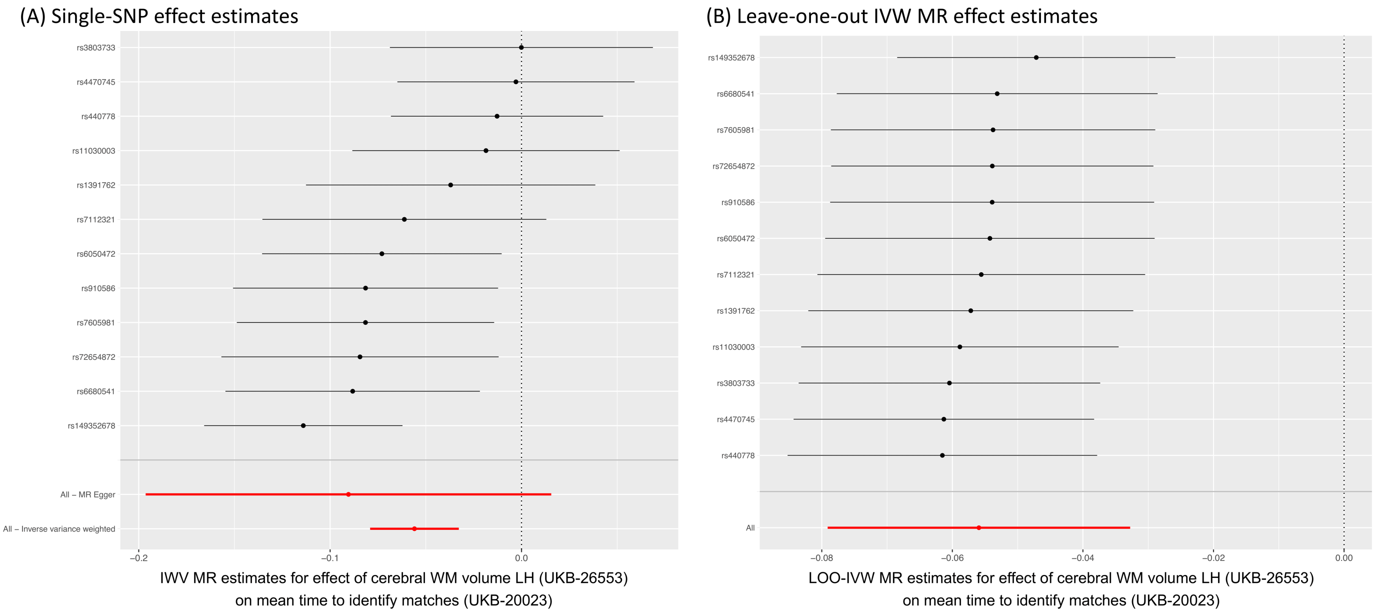


Figure 18: MR sensitivity analysis of the potentially causal effect of cerebral white matter volume (UKB-ID 26553) on correctly identifying matches (UKB-ID 20023). (A) Single-SNP ratio estimates. (B) Leave-one-out MR results. Errorbars indicate 95% confidence intervals.
